# Self and the Brain. The Immune Metaphor

**DOI:** 10.3389/fpsyt.2020.540676

**Published:** 2020-09-29

**Authors:** Silvia Sánchez-Ramón, Florence Faure

**Affiliations:** ^1^ Department of Clinical Immunology, IML and IdISSC, Hospital Clínico San Carlos, Madrid, Spain; ^2^ Department of Immunology, ENT and Ophthalmology, Complutense University School of Medicine, Madrid, Spain; ^3^ INSERM U932, PSL Research University, Institut Curie, Paris, France

**Keywords:** neurologic self, immune self, neurogenesis, schizophrenia, autophrenic disease

## Abstract

One of the fundamental questions in neuroscience is how brain activity relates to conscious experience. Even though self-consciousness is considered an emergent property of the brain network, a quantum physics-based theory assigns a momentum of consciousness to the single neuron level. In this work, we present a brain *self *theory from an evolutionary biological perspective by analogy with the immune *self*. In this scheme, perinatal reactivity to self inputs would guide the selection of neocortical neurons within the subplate, similarly to T lymphocytes in the thymus. Such self-driven neuronal selection would enable effective discrimination of external inputs and avoid harmful “autoreactive” responses. Multiple experimental and clinical evidences for this model are provided. Based on this self tenet, we outline the postulates of the so-called autophrenic diseases, to then make the case for schizophrenia, an archetypic disease with rupture of the self. Implications of this model are discussed, along with potential experimental verification.

## A Journey From Consciousness to Cell Biology

Since the philosopher David Chalmers raised “the hard problem of consciousness” (1), referring to the interrelationship between brain activity and the content of conscious subjective experience, there have been numerous attempts to explain consciousness from a scientific approach. Innovative approaches have arisen from neurophenomenology (2), functional neuroimaging tools (3) and from cognitive and evolutionary psychology (4). Through most of the Western science, consciousness has been considered as an emergent brain process: the result of the integration of either global neural synchronization (5, 6), or of many asynchronic microconsciousness (7), whereas a role for the single neuron unit is left aside. However, based on quantum physics, it has been hypothesized a cell-based theory that located the momentum of consciousness within the neuron microtubules (8, 9). Yet, it has been largely unexamined the question of a cell-based consciousness from a biological viewpoint underlying microcircuitries (10).

Here, we explore the gap between self-consciousness and neural activity from an evolutionary biological perspective (11). There is a fundamental ontological assumption that the central nervous system (CNS) evolved along the immune system (IS) by natural selection to better define individuals’ identity and interactions (12–14). We formulated the hypothesis that the distinction of self/nonself (internal/external) inputs by the brain has a cell basis similar to the immune self. The immune self/nonself recognition enables immune effective function without jeopardizing tissue integrity (15). We inferred this *self* brain hypothesis by analogy of basic principles between these two complex network systems, although through divergent processes and scales. Contemporary systems biology aims to understand the rules governing dynamic regulatory networks across different scales (genes, RNA, proteins, cells, systems, metasystems, organism). Self-organization underlies a generic property of many complex networks and of gene regulatory networks in particular, which control cell ontogeny (16, 17).

Comprehensive understanding of immune physiology has often been anticipated by clinical immunopathology. For instance, autoimmune diseases in vertebrates are induced by failure or imbalance in recognition of the tissues of the body itself. However, autoimmunity or low level self-reactivity is essential for normal immune response. Within the CNS, an inappropriate reactivity to self inner signals would risk of an “*horror autotoxicus*”^1^ (18) severely interfering with any cortical sensory, motor, and cognitive processes and therefore with consciousness. Inappropriate reactivity to self may characterize what we have called autophrenic diseases [from Greek αµτo- (*auto*), “self” and φρήν *(phrēn*), “mind, brain”], including epilepsy or schizophrenia (SZ). According to this novel view, epilepsy may represent excessive reactivity to self^2^, defined by sudden firing of a subset of neocortical neurons that causes unrealistic sensations (visual, olfactory, gustatory, auditory), involuntary jerking and altered self-consciousness (19). In the case of SZ, it would occur a dysfunction of subsets of neocortical neurons that discriminate *self* body signals as nonself, resulting in the rupture of the psychoneurological self (20, 21). Indeed, false fragmented perceptions are lived as a real self/nonself conflict and severely affect the patient’s awareness. These pathologies might suggest a role for the self at the brain subscale microcircuitries or even at the cellular level.

Immune self/nonself discrimination is instructed mainly during embryogenesis and early in life, through selection processes resulting in massive cell death that ensures efficient and highly specific response and eliminate potential highly auto-reactive lymphocytes. The principles guiding brain cortex functional organization during embryonic neurogenesis have not been fully investigated. If, following our argument, that were the case that a *self* principle governs brain function, such instruction in *self* should be developed during neurogenesis. Based on theoretical grounds and biological observations, we hypothesized that self-driven selection processes based on neuron connectivity occur within the brain cortex (11). In this article, to translate our hypothesis into a model, we firstly recapitulate well-established immune principles to hypothesize similar brain solutions at the system level in a unified evolutionary account; and secondly, we present a body of arguments to prove the plausibility of the hypothesis based on clinical and experimental observations. We then expand the analogy to outline the postulates for autophrenic diseases by extrapolating the postulates that define autoimmune diseases.

### Evolutionary Immune-Based Model for Brain Memory

The IS comprises at least two inextricably intertwined orders of recognition and memory that were developed through evolution: innate and adaptive immunity, the latter initially acquired in jawed vertebrates. Innate immunity displays a broad and pseudospecific quick and transitory response to stimuli (trained memory), essential for the survival of the organism as it is the only immunity present in invertebrates and plants. Adaptive immunity serves a highly specific and controlled response to a given antigen, and accounts for a long-term memory that, upon an ulterior *rendezvous* with this antigen is translated in a more accelerated, focused, and heightened response (22). Adaptive specificity stems from a bewildering diversity of unique T and B cell receptors repertoire, representing thus a huge qualitative jump in fine specificity to a changing environment by increasing complexity and efficiency of the response. Interestingly, innate immune cells (antigen presenting cells) drive antigen-specific memory activation and clonal selection of T lymphocytes’ pools.

We built on the similarity of this functional organization with the vertebrate CNS looking side by side innate/implicit and adaptive/explicit orders of recognition and memory (23). Through these analogies, our model challenged the traditional view of the hippocampus only ascribed to the explicit/adaptive memory by an innate/implicit memory structure and essential mediator to explicit (declarative) memory. Although the hippocampus and adjacent structures of the medial temporal lobe (MTL) share function homology to the mushroom bodies of invertebrates (such as insects and crustacea) (24–26) and are evolutionarily ancient brain regions, the involvement of MTL in short-term associative memory is recent (27, 28). Hence, MTL bridges both implicit and explicit processes, which is not a new idea, but in line with kinetic models (29), functional neuroimaging studies (30, 31), and processing-based models (32). Explicit/adaptive memory is founded in an overwhelming diversity of neocortical neurons spatially and dynamically arranged to cope with extremely precise recognition that enables high cognitive abilities. Similar to the IS, “innate” brain regions (MTL) guide the formation of long-term and highly accurate explicit/adaptive memories through specific synapses between ensembles of neurons (engrams) within the neocortex (33). The proposed classification of brain memory based on a singular immune standpoint opens up a broader evolutionary insight on the role of the MTL in implicit and explicit memory strategies of encoding, storage, and retrieval (23). For instance, the widely studied patient H.M., with extensive bilateral hippocampal lesion, is an eloquent case supporting that highly specific (explicit/adaptive) memory resides within the neocortex, and corroborates the psychological distinction between short- and long-term memory (34).

Our epistemological approach explores recognition and memory brain systems by an immune-based analogy to better align the functional network architecture within an evolutionary context. This standpoint has not been previously undertaken but to applying IS principles into artificial intelligence computation (for instance, data encryption and storage, intruders’ detection and recall algorithms, information rate efficiency, machine learning, etc.) (35, 36). Depicting similarities in basic biological principles between the IS and the CNS does not mean diminishing their gross differences or pretending to tackle the self, which lies beyond the boundaries of cell biology. In this essay, we intend to offer a new perspective on how brain emergent properties may be constrained by cell biology.

### Instruction in Self: Thymopoiesis Meets Neurogenesis

The IS and the CNS constitute two complex network systems in open exchange that sense and respond to the environment while preserving the identity and integrity of the organism (homeostasis). This information processing function requires the discrimination between a dynamic self (internal signals and symbiotic interactions) and nonself (i.e. the outer world and the other), property shaped through evolution up to a degree of high specificity (37). In addition to higher specificity and specialization, evolution has led to an ever-increasing complexity at genetic, molecular and cellular regulatory interactions (complementarity, positive and negative feed-forward and feed-back loops) to determine overall response within each system. It can be asserted that the specificity of T lymphocytes and neocortical neurons as a whole delineate our identity (immunological and cognitive) as individuals, which is mostly acquired during embryogenesis and shortly after birth. Hence, each one of us is equipped with a unique repertoire of T lymphocytes and neocortical neurons (38, 39) to face the external world early in life. These cells will be thereafter selected by novel stimuli from the external world through connections with innate cells/structures, triggering activation of cells ensembles; and the repertoire of lymphocytes and neurons will be then shaped according to external experience (40). The whole process underpins the extraordinary plasticity of these systems and the concept of individual history. Dealing with the notion of time is indeed an intricate issue of the IS and the CNS, which has equally fascinated physicists, biologists, and philosophers approaching the brain in particular. To put this temporal relationship in plain terms, these paradoxical “anticipatory” specific cells (T lymphocytes, neocortical neurons) can be selected at any given moment by external cues to become “past” memory cells ensembles and then travel forward to “the present” -now- during recall, while being key to modifying the “future” behaviour of the organism (definition of cognition).

The anticipatory repertoire of T lymphocyte receptors (TCR) is generated by combinatorial gene rearrangement within the thymus (thymopoiesis). T-lymphocytes subsequently undergo a multi-step process of selection in response to self (41–43). Those T lymphocytes that weakly respond to self stimulus (antigen) happen to survive, while those that do not react with self die, (positive selection), and those that react too strongly with self are most of them eliminated (negative selection) or preserved as regulatory T lymphocytes (44, 45). Thus, the self principle governing thymopoiesis determines the future immune response. As Janeway’s proverbial assertion dictates, “the immune system evolved to discriminate infectious non-self from noninfectious self” (43). Thus, autoimmunity (low level autoreactivity) is an inherent constituent of immune homeostasis, meaning that all peripheral T cells are self-reactive (46). The process described above is greatly simplified to focus on general mechanisms, and even if TCRs are highly specific, restriction of antigen presentation by the major histocompatibility complex (MHC) further increases the diversification of the individual response and is subjected to relative degeneracy.

How does the exquisite brain organization contribute to effective self/nonself recognition? And moreover, can we infer that there is a role for cognitive self at the neuron (engram) level? The underlying principles and mechanisms generating the functional specificity and diversity of neocortical neurons are far from being well known.

In a recent work, we suggested proceeding from an immune angle to address these questions and testing its validity or refutability. From this standpoint, we positioned the *self* as the axis of cortical neurogenesis (11), which would allow normal brain functioning and prevent costly autoreactivity. In parallel with the immune “logic”, we sustained that the brain evolved to discriminate perceptible non-self from non-perceptible self. Neocortical neurons selection would be guided according to the degree of self recognition, by which too low and too high self-reactive neurons would undergo programmed cell death. Such self-driven neuronal selection would remove neurons exhibiting none or excessive reactivity to self signals. As a result of this selection process, neurons exhibiting low self-reactivity would discriminate any novel external stimulus, fundamental for effective neuronal response, and tolerance induction in the neocortex (11). This would also mean that all neocortical neurons are somehow self-reactive. Interestingly, neocortical neurons are not only “perceptive” of the outer world but highly interconnected *via* associational projections to fulfil the needs of the organism by using internal and external information.

Neurogenesis is written in chemical and electromagnetic language, whose code has been explored from multiple perspectives. We present below a body of arguments to test the presented hypothesis based on experimental and clinical observations, notwithstanding the obvious limitations derived from current gaps in knowledge and to methodological barriers^3^.

- Internal cues during neocortical neurogenesis primarily instruct neuronal selection:
  -The most prevailing model for the development of neural circuits (Hebbian plasticity) states that synaptic connections are strengthened by correlated activity between pre- and post-synaptic neurons, while weakened by uncorrelated activity or lack of activity. Neural activity-dependent regulation is involved in cell type specification, dendritic branching, synaptic maturation and learning and memory through a complex program of gene regulation (47). However, this theory does not explain to date whether and how activity-dependent mechanisms sort out neurons during neurogenesis. Two sequential waves of programmed cell death (PCD) regulated each by two distinct gene programs occur at the cortex subplate during embryogenesis and early postnatal life: a first wave at ventricular (VZ) and subventricular zones (SVZ) that purges up to 70% of progenitor cells showing spontaneous voltage-dependent activity evolving to synchronized small networks; and a second wave that further selects around ≈30% of mature neurons at postmitotic zones once coherent neural circuits with thalamocortical and cortico-cortical connections have been established [reviewed in (48)]. According to our model, to delete neurons by internal (self)-reactivity criteria, which goes beyond neuron-quality control, the whole process evokes striking similarity with the two independent PCD waves of positive and negative selection of lymphocytes during thymopoiesis in: developmental timing (embryonic and postnatal); stepwise functional segregation according to cell activity (primarily of inactive progenitor cells and of synaptically-driven maturing neurons afterwards); cell specification and migration-maturation gradient; balanced specific excitatory and inhibitory cell subsets (49–52). We suggest that PCD purges primarily those neurons that do not show a reaction to internal self-signals and afterwards eliminates those that overreact to these internal signals (11).
  -*In vitro* and *in vivo* findings support that endogenous spontaneous firing rates at the neocortex may guide PCD waves during neurogenesis (53, 54). It remains unclear whether this neural activity functions in an instructive or in a permissive way, that is, if there are specific patterns of neural activity leading the neuronal fate; or in the contrary, it is that the mere presence of neural activity is sufficient for the neuronal survival. In fact, it has been described a rich repertoire of organized spontaneous activity patterns within the neocortex *intra utero* and perinatally, whereby depolarization of transmembrane voltage potentials above a certain threshold affects neurons survival and network organization (52, 55–57). By analogy to the presentation of the wide array of body self antigens (ectopically) to lymphocytes during thymopoiesis for *self* instruction, we hypothesize that the neurogenesis harbors the huge cast of *self* electrical signals that will instruct and select by PCD the repertoire of neocortical neurons. If this is so, the search of promiscuous gene expression within the cortex of those *self* electrical signals to promote self tolerance—the equivalent to the autoimmune regulator (AIRE) gene by thymus stroma—would be of great interest. We have not found experiments addressing specific-spike series or specific molecules within the SVZ/subplate in relation with differential neuronal sorting and circuit formation that may explain specific pattern-dependent regulation in neuronal segregation.
  -Experimental models of cortical neurons xenotransplantation may shed light on the issue of a potential host *self* brain instruction. After single human pluripotent-stem cells (PSC)-derived cortical neurons xenotransplanted to the neonatal (P0/P1) mouse brain into the subplate, neurons integrated in the mouse cortex. Around 17% of transplanted single neurons matured and displayed responses to sensory stimuli that resembled those of the host neurons, stressing the specific nature of the circuitry. The authors suggested that the host brain provided not only permissive environment but also instructive cues regulating precise circuit formation (58). The host *self* instruction in human neurons seemed to be restricted during maturation at subplate by presynaptic partners (thalamus or cortex). The experiment was performed in neonatal mice, showing ulterior fine-tuning to external stimuli. By contrast, very limited synaptic integration results when the PSC-derived human cortical neurons were transplanted in bulk into the mouse cortex, which are less accesible to receive inputs from the host brain (59).
- The presence of specific excitatory (E) and inhibitory (I) neuron subsets within the neocortex assures homeostasis and functionality.
  -A cortical organoids model from induced-PSC generates E and I neurons, i.e. glutamate and gamma-aminobutyric acid (GABA) neurons, respectively, which account for the generation and maintenance of oscillatory activity and synchronization of the network. Small-scale functional electrophysiological networks by these neurons subsets coordinate information flow resembling preterm neonatal brain activity (60). Inhibitory neurons act as a necessary “self-check” for excessive or prolonged responses by which the cortex precisely regulates functional effective connectivity. Impaired E/I balance is associated with several diseases, such as epilepsy and SZ.
  -Stimulus-specific E and I assemblies have been described in the ferret primary visual cortex (61) and posterior parietal cortex of mice (62), pointing to selective inhibition by GABAergic neurons, similarly to antigen-specific regulatory T cells, controlling excessive responses and maintaining homeostasis and tolerance to self.
  Recent work has shown that positive selection of I neurons (early postnatally) occurs and is coordinated by activity-dependent connections to E neurons (63, 64). The generation of the combinatorial code of unique neuron-tag molecules, such as protocadherins (65), seems to regulate a critical window of PCD of cortical interneurons (66). Adequate balanced networks of I and E neurons is adjusted by consecutive waves of PCD (48, 63)-. This phenomenon further supports the concept of neuron-specific selection by *self* E neurons, providing an evolutionary advantage for the rapid increase in pyramidal neurons in the primate lineage (63).
- Lesion studies can yield valuable information about the putative contributions of neural selection *in utero* to cortex functionality.
  -Malformations of the cortical development (MCD) may be due to a broad array of disorders that disrupt the tightly spatiotemporally orchestrated process of neurogenesis (proliferation, migration, differentiation, synaptogenesis, apoptosis, synaptic pruning). MCD may affect the neuronal pool and connectivity of specific circuits, causing a wide spectrum of cognitive deficits, seizure disorders or neurospychiatric diseases, such as schizophrenia or autism (67). Depending on the time and degree of the neurodevelopmental insult, clinical onset can be delayed (latency) thanks to compensation mechanisms through E-I interactions or other plasticity mechanisms, or to the time lapse to acquire a task that relies upon the specific neurobiologic substrate. A main feature is thus the fine regional and functional specificity of the affected neocortical neurons, which translates into hyperexcitability (anti-self) interfering their related circuits.
  -The self brain hypothesis can be fully integrated into the programmed changes described for differentiation and maturation sequences of cortical neurons during neurogenesis and also provides a new dimension to the whole biological process. Defects or interferences in these developmental changes would result in excess of specific autoreactive E neurons, contributing to the hyperexcitability and ultimately in epilepsy. The view we present here might add conceptually important elements to the understanding of epileptogenesis. Accumulative or dysbalanced action potential firing of individual autoreactive neurons may disrupt the ensemble of specific circuits. The earlier the insult, the more severe or intractable the disorder, with persistent deleterious effects despite the high plasticity of the immature brain (68, 69). Subtle alterations on electrical activity during neurogenesis affect neuronal segregation and connectivity, and can cause many forms of epilepsy. These observations may suggest that specific features of neural activity (rather than just the presence of neural activity) are important for the neuronal selection during neurogenesis, pointing to the hypothesis that endogenous specific neural activity is instructive for neuronal selection within the neocortex. This fact may also reflect the purging function of non-reactive or highly self-reactive neurons. Our theory may also provide the basis for therapeutically significant avenues of development. Currently available pharmacological treatments of epilepsy are mainly symptomatic, none is curative or preventive. Moreover, anticonvulsivants show suboptimal effectiveness with long-term detrimental neurologic effects. New functional tools based on specific cellular resolution biomarkers to identify the hyper-reactive E neurons populations may favour new therapeutical interventions to selectively block these circuits. Also, these neuronal resolution biomarkers could propel the dissection of specific circuits and hopefully the development of new drugs based on pathophysiological mechanisms.
  -Gene lesions associated to control of apoptosis during neurogenesis underlie several types of MCD, resulting in epileptogenesis when not to perinatal lethality (70). Given that genes and epigenetic modifications regulating the survival of specific populations of neurons are now beginning to be elucidated, advances within the field will foster progress in understanding cortical neuron segregation and neural circuits during neurogenesis (48, 71).
  -Murine models of MCD, in which targeted chemical and physical insults during early development within the SVZ induce pronounced cortical hyperexcitability and reproduce the pathological and clinical findings of congenital forms of epilepsy [reviewed in (72)]. Timing and location (region and layer) of the induced lesion are key to the MCD clinical expression, suggesting that specific alteration of neuronal seggregation processes lead to hyperexcitability and altered connectivity.
- Our hypothesis challenges the currently accepted alternative hypothesis of “instruction from external inputs”:
  -In the auditory system, the selection and wiring of neocortical neurons within central sensory areas precedes the formation and priming of sensory receptors, circuits that will be refined later on by external inputs (73, 74). Before hearing onset, the precise temporal pattern of spontaneous pre-hearing activity is crucial for the formation of precise tonotopy in the central auditory pathway, supporting the role of self-instruction orchestrated development.
  -Extreme examples or experiments of nature, such as complete unimodal sensory deprivation or anophthalmia (bilateral congenital absence of eyes) may give relevant insight into this issue. The connections patterns of organization in the cortex visual areas in the absence of retinal waves and visual experience of anophthalmic patients are not significantly different from normal sighted individuals (75–77). This finding may suggest that the visual retinotopic architecture of the neocortex does not primarily depend on external sensory instruction, but that *in utero* neural activity primarily shapes functional properties of cortical networks (75, 77).

**Figure 1:**
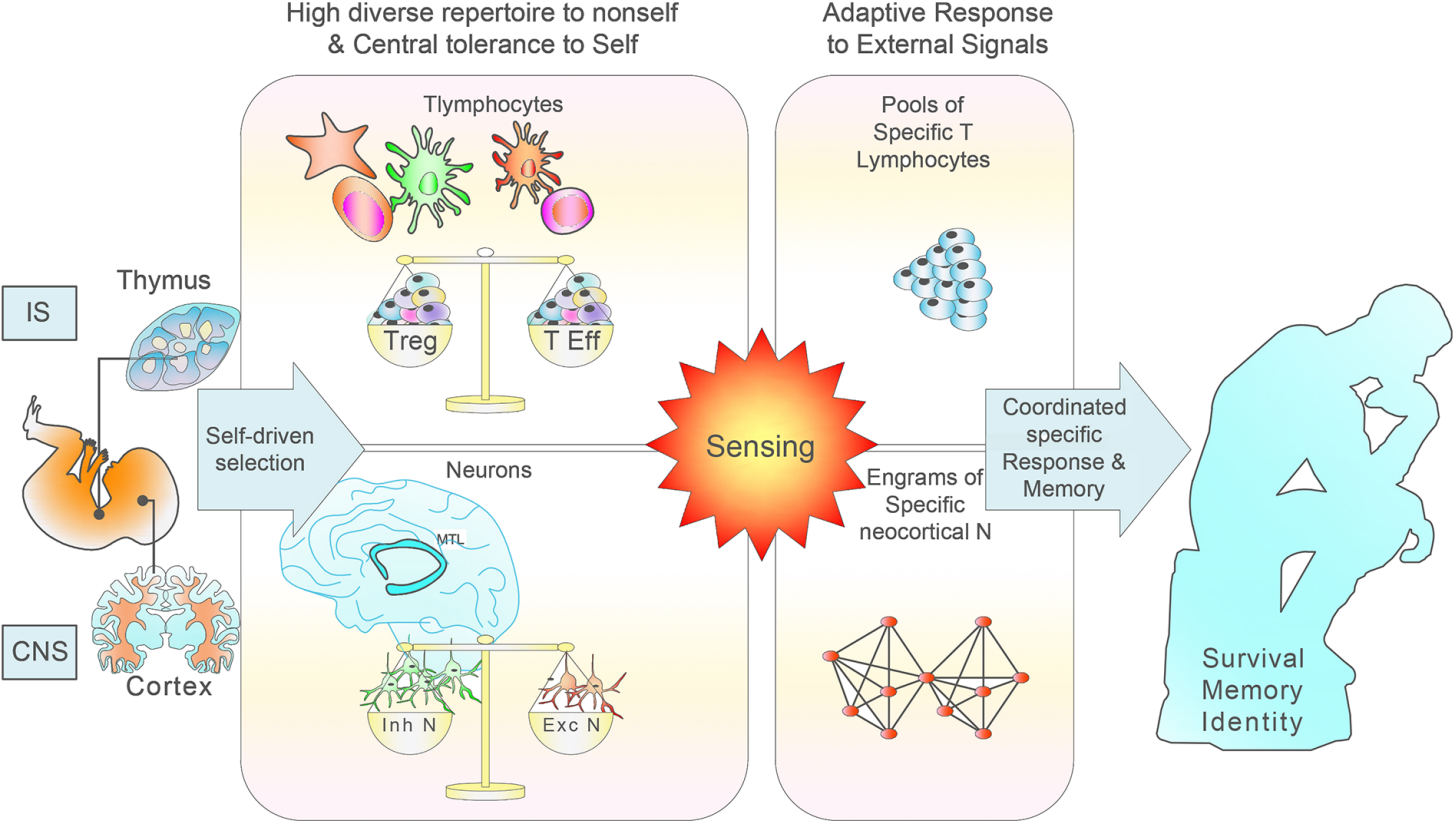
Scheme of the normal IS and CNS development, maturation and functioning based on self education. During the generation of T lymphocytes in the thymus, it occurs a multi-step process of selection in which the great majority of highly self-reactive T cells die, leading to an immunocompetent and self-tolerant pool of naïve T lymphocytes. Clonal deletion is incomplete, as many self-reactive T cells find their way to the periphery where they constitute a constant risk for the development of autoimmune disease. During neurogenesis, the effective removal of most autoreactive neurons relies on a coordinated multi-step selection, where most low reactive neurons to self would be able to discriminate external signals. A number of selection mechanisms is of fundamental relevance for neocortical neuron development, responsiveness to external signals and tolerance induction to internal signals. Matching of electrical signals through sequential layers allows combinatorial signals with increasing complexity in a unique and extremely precise way.

Therefore, from many directions we find support for the working hypothesis that self/nonself discrimination is the result of a biological process primarily instructed from early neurogenesis by host self signals, to build an extensive repertoire of neocortical neurons. In both the IS and the CNS, each post-selection repertoire would thus represent, respectively, a mirror image of the immune and neurological reality that we are able to sense and with which we can constantly interact (**Figure 1**). This primary neuronal repertoire and neural circuits will be secondarily refined by external inputs during development, an activity-dependent process that is plastic. The entire process would endow the brain with a cell basis for consciousness and hence self-consciousness.

## Conceptual, Clinical and Experimental Arguments of Autophrenic Disease. Revisiting Schizophrenia

In order to formulate a general scheme and a case example, we will firstly take advantage of the well-established criteria defining autoimmune disease (AD) (78) to draw the principles of autophrenic disease in order to evaluate the self model in brain pathology (**Table 1**). Secondly, we will apply these principles to predict SZ pathophysiology. ADs are multifactorial conditions that result from the complex interplay of risk and protective factors, in which autoreactive T lymphocytes induce specific tissue damage or dysfunction. Intrinsic (genetic, epigenetic, endocrine, and psychoneurological), extrinsic (environmental), and stochastic factors induce cumulative effects that eventually lead from health to illness, with a waving course. A key pathophysiological role of innate immunity in AD has been unveiled (98). An interesting phenomenon is how disparate AD share immune mediators (autoimmune tautology) (99), while differ in the target tissue or organ, highlighting that specificity is given by the antigen-specific autoreactive T lymphocytes. A pro-inflammatory/anti-inflammatory cytokine imbalance favours differentiation and amplification of these target-specific autoreactive T lymphocytes.

**Table 1:** Model of autoimmune disease criteria proposed by Rose and Bona based on 1957 Witebsky’s postulates (78).

	AUTOIMMMUNE DISEASE	AUTOPHRENIC DISEASE
**Concept**	- Excessive or inappropriate adaptive immune response against the antigens of the body itself (autoAg) - Loss of tolerance to autoAg - Tissue damage and/or dysfunction, chronic inflammation	- Excessive or inappropriate specific excitability against the neural signals of the body itself - Loss of tolerance (inhibition) to self-signals - Alteration of specific cortical region architecture and function (local and distant)
**Direct evidence**	- Activation of autoreactive T lymphocytes or autoAb targeting Ag-specific tissues or organs	- Disease-specific highly autoreactive excitatory cortical neurons and engrams at specific brain regions (hyperactivity of sensory cortices during hallucinations) inducing disrupted connectivity and cortical dysfunction (79–83) - Cortico-subcortical hyperconnectivity within sensorymotor areas, while reduced PFC-thalamic connectivity (84)
	- Disease-specific autoAb inducing dysfunction (cell damage, binding to inhibitory or stimulatory receptor or enzyme or hormone)	
	- Replication of disease by passive transfer of pathogenic autoAb/autoreactive T lymphocytes	- Replication by disinhibitory action of NMDAR antagonists (i.e. ketamine) through blockade of E-to-I synapses
	- Proliferation of T lymphocytes *in vitro* in response to autoAg	- Hyperactivation of neurons in response to self-produced sensory stimuli (85)
	- Induction of disease by xenotransplantation of human target tissue plus sensitized T lymphocyte to severe combined immunodeficient mice	
	*- In vitro* cytotoxicity of T lymphocytes towards cells of the target organ	
	- Desensitization with low dose and repeated exposition to autoAg	- Beneficial effects of non-invasive brain stimulation, such as slow rTMS (86) and direct stimulation on auditory hallucinations and negative symptoms refractory to antipsychotics to reduce brain excitability (87)
**Indirect evidence**	- Genetically induced disease models - Experimental immunization or animal models of spontaneous autoimmunity	- Genetically induced disease models: abnormal patterns of oscillatory activity and firing in PFC (88), but not HP in DISC1 knock-down mice (89) - Experimental SZ by somatostatin interneuron dysfunction at PFC (90) - 3D neural tissue model organoids 15q11.2 microdeletions (91) - AutoAb targeting neuronal antigens that disrupt synapsis and cause functional dysconnectivity in a subgroup of SZ (92, 93).
	- AutoAb located at the site of lesion (as well as immune complexes)	- Aberrant anti-self-firing synapses at affected regions (disturbed gamma band synchrony) (94)
	- Adoptive regulatory T cell therapy in autoimmune diseases (95)	- Grafting of GABA-ergic progenitors can reduce seizures and psychosis (96)
**Circumstantial evidence**	- Association with other autoimmune diseases. - Shared mediators (AD tautology)	- Association with other autophrenic diseases: epilepsy (9% to 52%) (97), depression, autism (30%), etc. - Shared mediators among different neuropsychiatric diseases.
	- High risk and protective HLA haplotypes, thymogenesis and other immune-related genes	- Main neuron-specific genetic signatures (neuronal connectivity and synaptic plasticity). - Protective HLA haplotypes
	- Lymphocytic infiltration of the organ, especially if there is a restriction in V gene usage	- Neuron-type-specific and cortical region-specific epigenetic linking genetic expression signature.
	- Favorable response to immunomodulation and immunosuppression.	- Favorable response to neurotransmitters’ inhibitors, anti-epileptic medications, lithium, electrostimulation (rTMS and transcranial direct or alternating current stimulation) and electroconvulsive therapy.

Ab, antibody; Ag, antigen, DISC1, disrupted in schizophrenia; HLA, human leukocyte antigen; E-to-I, Excitatory-to-Inhibitory; HP, hippocampus; NMDA-R, N-Methyl-D-Aspartate receptors; PCF, prefrontal cortex; rTMS, repetitive transcranial magnetic stimulation.

Autoimmune diseases in strict sense must fulfil at least three criteria of direct evidence and most of those of indirect and circumstantial evidence. We show autophrenic disease postulates in parallel and specifics applied to the case of schizophrenia.

**Figure 2:**
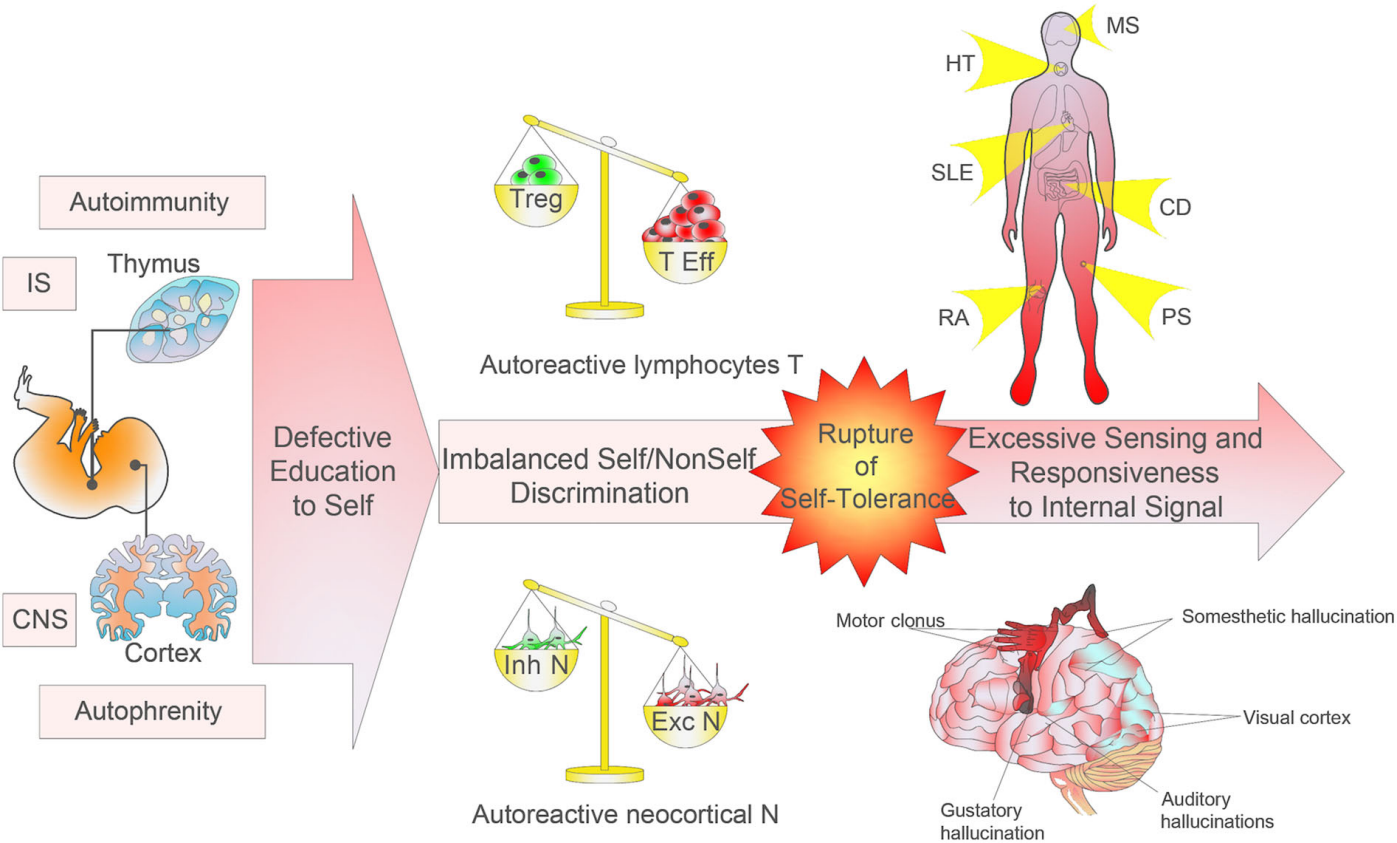
Imbalanced self/non-self education and discrimination. The research on monogenic autoimmune syndromes has shown the relevance of mutations harbored by defined proteins of thymic selection processes and the development and activation of regulatory T cells. Environmental factors operating in a genetically susceptible host may trigger or exacerbate the pathological autoimmune process, induce mutations in genes coding for immunoregulatory factors, or modify immune tolerance or regulatory and immune effector pathways. Autoimmune diseases can affect any site and any organ system in the body. In parallel, monogenic autophrenic diseases are related to protein mutations involved in cortical neurogenesis, synaptic plasticity and connectivity, and chromatin remodelling in specific cortical areas or functions, which can affect any body’s function. The neocortex shows an extremely fine topography and plays essential functions of sensory, motor, and cognitive processes. The autophrenic diseases so conceived may provide a better understanding of the complex neurobiology of neurodevelopmental disorders. Although the autoimmune/autophrenic diseases affect specifically adaptive/explicit recognition, the pathophysiology is induced by alteration in evolutionarily more ancient cells constraining adaptive response (i.e. antigen presenting cells and the limbic system, respectively).

On the basis of this proposed neurobiological self model, the nomenclature autophrenic disease designates complex multifactorial diseases in genetically susceptible individuals, modulated by endocrine and immunological factors, as well as psychological events in life (11). They define excessive or aberrant responses of autoreactive excitatory cortical neurons to specific endogenous neural inputs and/or defective inhibitory neurons, which disrupt certain cortical brain structures and functions (**Figure 2**). Similarly to autoimmune tautology, a cortical E-to-I neurotransmitter imbalance characterizes autophrenic diseases. By analogy with autoimmune pathogenesis, evolutionarily ancient brain structures (in particular the limbic system) would be expected to play a primary role. To complicate the scenario even further, autophrenic disease may be triggered by autoimmunity, as autoreactive T lymphocytes can target specific neurons’ subsets (92, 93).

SZ is a prevalent mental disease characterized by a severe and disabling course in which the rupture of the psychic self is nuclear to the disease (100). Endocrine factors, such as male predominance, clinical onset in adolescence or early adulthood, and worsening at postpartum may play a part, coincidentally with cognitive maturation of the prefrontal (PFC) and parietal cortices (101, 102). Foetal and early childhood immune priming, such as maternal infection or active brain inflammation, are strongly associated with susceptibility to disease (103). Cumulative evidence across different experimental approaches (copy variant numbers, rare and *de novo* variants, genome wide association analysis, transcriptome and 3D genome structures) has restored its original conception as a neurodevelopmental disease, which stretches the way back to neurogenesis during embryonic development (88, 104, 105). SZ shows strong heritability estimated from twin studies of 79% (106, 107), while is highly polygenic with very low individual impact. Main mutations involve synaptic connectivity and chromatin remodelling (108). Transcriptome analyses of epigenetic regulated genome have revealed specific cell-type-dysregulation in the frontal lobe of SZ patients (109–111). In addition, a role of activated microglia during neurogenesis that affect neuronal segregation and connectivity has been hypothesized (112, 113). It is postulated that SZ is a heterogeneous large scale dysconnectivity syndrome (114). According to age of onset, SZ has been classified in a rare but severe childhood form with widespread cognitive impairment; and an early adult form with predominantly PFC-related verbal and executive abilities decline (115). Hallucinations and passivity phenomena (delusions of alien control), with disrupted discrimination between the external and internal inputs, are cardinal to SZ. In particular, auditory (audible thoughts, voices arguing and commenting about the patient in third person), visual and cenesthesic hallucinations are common (first rank symptoms). Overactivity in primary and secondary sensory areas seem to be involved in aberrant gamma oscillations to endogenous inputs from pyramidal neurons (83) despite absence of actual external stimuli (81, 82, 85). Some authors have explained this aberrant neuronal firing to inner signals in terms of feed-back loops and distant connectivity involvement with excessive expectation, by which altered recognition of endogenous signals were misattributed as coming from an external source (79). Experimental evidence suggests that SZ patients show similar or even outperform healthy controls on visual discrimination skills. More difficult is to explain negative symptoms (e.g., alexithymia) in terms of *self*, which might be understood by local and long-scale brain connectivity disturbances (80, 114).

The E/I imbalance is considered key for SZ pathophysiology (116). However, the non-uniform distribution of the E/I imbalance—such as distinct hyper- o hypodopaminergic (117) and hyper- or hypogabaergic brain regions and cortex areas (118)—suggests their secondary role in SZ pathophysiology. This uneven distribution dampens the therapeutic efficacy of current drugs. A profound defect in inhibitory GABAergic interneurons has long been established as the most common finding (119). GABAergic defect is region-specific, with decreased expression of the neurotransmitter GABA, decreased ability to generate gamma oscillations, decreased GABA receptors and low inhibitory neuron markers at the basal ganglia, the visual cortex and in the cerebrospinal fluid (120). In contrast, GABA is increased at PFC in unmedicated patients (188, 121). Excessive dopamine and glutamate (due to hypofunctioning N-methyl-D-aspartate receptors) within the hippocampus and striatum (79, 122), account for overactivity of the primary and secondary sensory areas that induce misperceptions, while both neurotransmitters are decreased at brain cortex. Conventional therapies have been directed to modulate the E/I balance, with partial effectiveness and potentially severe side-effects.

We suggest that E/I imbalance arises from dysregulation between self-specific excitatory and inhibitory neurons (or synapses) at circumscribed cortical areas. Involvement of the cortical areas responsible for specific recognition and explicit memory (primary and secondary sensory areas) might underscore a perceptual basis of self-consciousness. Thus, the dysregulation between self/nonself-reactive neurons would be the key event in the autophrenic disease, with subsequent local E/I imbalance and altered connectivity processing and metacognition. In this context, our theory advocates for the identification through single-cell resolution biomarkers of specific hyper-reactive neurons and the ensemble of their circuits as a new means for better understanding these diseases and for the exploitation of more specific blocking strategies. Alternativelly, targeting specifically GABA inhibitory neurons at the former circuits might be effective to control hyperexcitability. Promising strategies targeting specific cell types are being advanced by calcium imaging (123). Selective optogenetic activation of individual cortical neurons can trigger relevant ensembles and modify behavioural responses in mice, supporting a causal link between the cortical neuron (*self*-reactive neuron in our view) and learned behavior (124, 125). In other order of strategy, a psychodynamic approach would be interpreted as a means to “desensibilize” autoreactive neurons by modifying the context of presentation of the input from MTL or basal ganglia.

## Discussion and Future Perspectives

The IS and the CNS are genuine self-referential systems. The analogies between the two systems for cell recognition and memory strategies may support that general principles are operating. In the IS, discrimination between self and nonself is based on certain criteria of reactivity to self (46). Autoimmunity is an implicit constituent of cell immune homeostasis. Its deregulation may lead to autoimmune disease. In this work, we present a cell brain *self *theory from an evolutionary biological perspective by analogy with the immune *self*. At the cell level, we postulate that the extreme precision of recognition and association that enables the brain to perceive, memorize, anticipate, and to act is primarily instructed by reactivity to self during early life, when main neocortical structures and circuitries are organized. The implicit assumption of the biologic brain *self* theory is that autophrenity is consubstantial to brain physiology and homeostasis. Early life individual environmental exposure is key to refine the functional structure by means of a trade-off between chance (contingency) and necessity (adaptation). As for autoimmunity, the concept of autophrenity extends to self-regulation within the network.

The actual role of the immune self/nonself principle to determine the outcome of the immune response is finely tuned to a spatio-temporal dimension. The response will depend not only in the antigen *per se* but in anatomic location (site-specific) and contextualization of the immune challenge (126). To illustrate the point made, if any antigen is delivered, how this antigen will be seen by the IS will be tuned by intrinsic (quantity, duration of exposition, location, etc.) and extrinsic factors that will differentially impact the response (effector against tolerant). Among the intrinsic factors, the valence, perceived at the system level as danger/reward or as discontinuity (surprise)/continuity (127), is key to the outcome and mainly driven by innate immunity. Likewise, the response of the CNS is not only determined by the stimulus *per se* but strongly conditioned by the context of presentation through innate structures (mainly the limbic system) as a danger/reward valence, by evolutionarily more sophisticated feelings (128) and by previous experience (129). This valence modulation has impressive therapeutic potential and is being exploited in strategies as, for instance, desensitization therapies in allergic diseases and by cognitive-behavioural psychotherapies (130), respectively.

The structure of cognition is metaphorical, built on pattern recognition at different scales (13). We endeavoured to explore the neurologic self as a metaphor of the immune self at the cellular level, which provides grounds for understanding complexity from another biological system logic. Through this immune analogy, our hypothesis provides a potential guiding principle, which may add both biologically and, likely, therapeutically significant avenues for development. The exploration by this approach of cortical neurogenesis might offer a bottom-up explanation of the system functioning as a whole, and a new insight into some neuropsychiatric diseases.

The present work addresses the autophrenic disease by analogy with autoimmune disease. Other approaches, such as computational phylogenetic analysis of homologue genes, which code for the receptor pathways of neocortical neurons across species, could add complementary experimental verification of the theory, which could trace the plausible evolutionary sequence. This theory constitutes the basis for current ongoing work.

SZ semiotics is already shifting to link mental phenomena with underlying neurobiological mechanisms (131, 132) given the overlap among psychiatric manifestations and diseases. As long as current theories about etiologically complex illnesses like SZ remain open, we hope our theory will help to change SZ understanding. Following this reasoning, our theory points at aberrant anti-self neuronal responses behind anti-self neuropsychiatric disorders in a more meaningful dimension from a biological point of view. The brain self theory opens a new conceptual reflection on the gap between biological and conscious self. In his incompleteness theorem, Kurt Gödel decried the incapability for any formal system to be proven or disproven from within the system. Given the inherent limitations to consistently approach the functioning of the brain from brain logic, our inferential perspective from an immune metaphor would argue for its consistency. The brain self theory is beheld by a number of biological, experimental, and clinical findings in SZ that deserve further investigation. Typical positive SZ symptoms like hallucinations may help to better understand how excessive self-reactive excitatory neuronal activity of the neocortex may compromise the discrimination between the external world and internal experience and so alter the structure and connectivity of affected areas and distant circuits.

We expect that an understanding of neurobiology in terms of *self* at a wide-system functioning will open new targeted therapeutic strategies in disparate anti-self brain diseases and hopefully inspire further investigation.

## Author Contributions

SS-R wrote the first draft. SS-R and FF have equally contributed to the conception, design, critical revision, and final approval of the manuscript. SS-R and FF agree to be accountable for all aspects of the work in ensuring that questions related to the accuracy or integrity of any part of the work are appropriately investigated and resolved.

## Conflict of Interest

The authors declare that the research was conducted in the absence of any commercial or financial relationships that could be construed as a potential conflict of interest.

## Footnotes

1 Paul Ehrlich proposed the term “*horror autotoxicus*” to designate the unwillingness of the organism to the production of autoantibodies “amboceptors directed against its own tissues”.

2 Hereinafter “self” alludes to biologic self (at cell level) although otherwise specified.

3 References to the original papers will be made when these provide essential contribution to the discussion.
